# Cytochrome *c* as a Potentially Clinical Useful Marker of Mitochondrial and Cellular Damage

**DOI:** 10.3389/fimmu.2016.00279

**Published:** 2016-07-20

**Authors:** Theodoros Eleftheriadis, Georgios Pissas, Vassilios Liakopoulos, Ioannis Stefanidis

**Affiliations:** ^1^Department of Nephrology, Medical School, University of Thessaly, Larissa, Greece

**Keywords:** cytochrome *c*, danger-associated molecular patterns, mitochondrial, apoptosis, necrosis

## Abstract

Mitochondria are evolutionary endosymbionts derived from bacteria. Thus, they bear molecules, such as mitochondrial DNA (mtDNA) that contains CpG DNA repeats and *N*-formyl peptides (FPs), found in bacteria. Upon cell necrosis or apoptosis, these molecules are released into the interstitial space and the circulation and recognized by the immune cells through the same receptors that recognize pathogen-associated molecular patterns, leading to inflammation. Other mitochondrial molecules are not of bacterial origin, but they may serve as danger-associated molecular patterns (DAMPs) when due to cell injury are translocated into inappropriate compartments. There they are recognized by pattern recognition receptors of the immune cells. Cytochrome *c* is such a molecule. In this review, experimental and clinical data are presented that confirms cytochrome *c* release into the extracellular space in pathological conditions characterized by cell death. This indicates that serum cytochrome *c*, which can be easily measured, may be a clinically useful marker for diagnosing and assessing the severity of such pathological entities. Reasonably, detection of high cytochrome *c* level into the circulation means release of various other molecules that serves as DAMPs when found extracellularly, the mtDNA and FPs included. Finally, because the release of this universally found compound into the extracellular space makes cytochrome *c* an ideal molecule to play the role of a DAMP *per se*, the available experimental and clinical data that support such a role are provided.

## The Mitochondrion as a Source of Danger-Associated Molecular Patterns

The idea that the immune system has been evolved to discriminate non-infectious self from infectious non-self revolutionized our thinking about its function and regulation (1). Matzinger extended this perspective even further and proposed that the immune system is designed to react to danger signals, i.e., to signals of cell destruction or tissue demolition (2). Later, pattern recognition receptors (PRRs) were discovered on the surface or inside the immune cells able to recognize pathogen-associated molecular patterns (PAMPs) present in phylogenetically distant microbes, or danger-associated molecular patterns (DAMPs), i.e., self molecules present in inappropriate compartments due to cell destruction (3).

Mitochondria are evolutionary endosymbionts derived from bacteria (4, 5), containing molecules that are normally present in prokaryotes and, consequently, under certain circumstances can be recognized by PAMPs’ specific PRRs eliciting an immune response. The best-defined mitochondrial DAMPs are the mitochondrial DNA (mtDNA) and the *N*-formyl peptides (FPs). Like bacterial DNA, mtDNA is characterized by the presence of unmethylated CpG repeats, recognized by the PRR toll-like receptor 9 (TLR9) (6). Injection of mtDNA intra-articularly induced a monocyte-dependent arthritis in mice, while mtDNA was also detected in the synovial fluid of patients with rheumatoid arthritis but not of control subjects (7). In an experimental model of injury-associated systemic inflammatory response syndrome, the role of the released mtDNA and its recognition by TLR9 in activation of polymorphonuclear neutrophils and establishment of this syndrome has been confirmed (8). Release of mtDNA and its recognition by TLR9 have also been incriminated in an experimental model of cardiomyopathy (9). Concerning FPs, it has been elaborated that they are encoded only by bacterial or mitochondrial genes due to initiation of protein synthesis with *N*-formylmethionine. FPs are recognized by specific receptors, such as the FP receptor 1 (FPR1) on human neutrophils, which by controlling neutrophils migration, activation, and degranulation is implicated in the pathogenesis of both infective and sterile inflammatory conditions (10). Utilizing the same experimental model of injury associated systemic inflammatory response syndrome as in the case of mtDNA, the role of the released FPs and their recognition by FPR1 in activation polymorphonuclear neutrophils and establishment of this syndrome has been validated (8). In experimental hemorrhagic shock or trauma, mitochondrial FPs were released into the circulation and recognized by FPRs on basophils inducing the systemic inflammatory response syndrome-associated hypotension. Circulating FPs were also recognized by FPRs on both mast cells and neutrophils inducing systemic inflammatory response syndrome-associated airway contraction and systemic inflammatory response syndrome-associated lung neutrophils infiltration, respectively (11, 12).

## The Potential of Cytochrome *c* to be a Mitochondrial DAMP or a Clinically Useful Marker of Cell Death and Release of Other DAMPs into the Extracellular Space

Cytochrome *c* is a small soluble electron carrier hemeprotein located in large amounts in the inner mitochondrial membrane. By transferring electrons from complex III to complex IV, cytochrome *c* facilitates cell energy production (13). Although is not encoded by mtDNA but by a gene located at the short arm of chromosome 7 (14), maintenance of the cytochrome *c* inside the mitochondrion is imperative, since its release into the cytosol results in cell apoptosis. During cell apoptosis cytochrome *c* is released into the cytoplasm where it binds and activates the apoptotic protease activating factor-1 (Apaf-1) allowing its binding to ATP and the formation of the ring-like apoptosome. Apoptosome through its caspase recruitment domain (CARD) binds, proteolyzes, and activates procaspase-9. Next, caspase-9 proteolytically activates effector caspases-3, 6, and 7, key players in the execution-phase of cell apoptosis (15). Reasonably, in the occurrence of cell damage, cytochrome *c* could be released into the extracellular space, where it may serve as a DAMP, since many self-molecules when are translocated into inappropriate compartments play such a role (16). Thus, according to the location of cytochrome *c*, anti-inflammatory or pro-inflammatory properties may be attributed to it. Under normal conditions, cytochrome *c* resides into the mitochondria. Exodus of cytochrome *c* into the cytoplasm induces the non-inflammatory process of apoptosis, whereas once translocated into the extracellular space it may trigger inflammation. In parallel, in the latter case assessment of cytochrome *c* in extracellular space could be used as a marker of severe mitochondrial damage and cell death. Reasonably, the release of cytochrome *c* into the extracellular space during cell death is accompanied by and indicates the release of various intracellular components, the mtDNA and FPs included, that can be recognized by immune cell PRRs eliciting an immune response.

Plenty of experimental and clinical data recapitulated the release of cytochrome *c* from dying cells either due to apoptosis or necrosis. In splenocytes, immediately after heat shock-induced necrosis or within 2 h after an apoptotic insult, cytochrome *c* was released into the extracellular space in an intact monomeric form (17). In experimental resuscitation of cardiac arrest, circulating levels of cytochrome *c* increased progressively to levels 10-fold higher than in sham rats and correlated inversely with survival (18). A study of drug-induced hepatotoxicity revealed that cytochrome *c* can be used as a marker of liver injury (19). In experimental and clinical acute kidney injury, cytochrome *c* was also detected in the plasma and urine (20). Interestingly, release of cytochrome *c* into the culture medium due to staurosporine-induced apoptosis of neuronal cells enhanced apoptosis further (21), and addition of exogenous cytochrome *c* in lymphocyte cultures induced apoptosis (22). This toxic effect of extracellular cytochrome *c* deserves further evaluation.

Besides experimental data, several clinical studies have confirmed release of cytochrome *c* into the extracellular space and, finally, into the circulation in various conditions characterized by cell death. Indeed, release of cytochrome *c* into the circulation has been confirmed in patients with myocardial infarction (23). Serum level of cytochrome *c* was found increased in patients with several liver diseases. Mean serum cytochrome *c* concentration was 187.1 ng/mL in patients and only 39.8 ng/mL in healthy controls. In these patients, cytochrome *c* level was correlated well with other biochemical markers of liver injury as well as with the necroinflammatory score and the apoptotic index of liver biopsies (24). In patients with acute liver failure, serum cytochrome *c* was elevated, correlated well with various biochemical markers of liver injury, the mitochondrial aspartate aminotransferase included. Mean serum cytochrome *c* concentration was 10,686 pg/mL in patients with acute liver failure and only 112 pg/mL in healthy controls. Its level also paralleled with the severity of hepatic coma (25). There are also several studies remarking on the possible role of serum cytochrome *c* as a prognostic marker in various types of cancer, with the higher levels showing high-turnover and, consequently, more aggressive tumors. On the other hand, elevated levels of serum cytochrome *c* after chemotherapy may be a good prognostic factor, indicating increased chemotherapy-induced cancer cell apoptosis (26–29). For instance, in patients with operable malignant tumors, median serum cytochrome *c* concentration was 20.6 ng/mL compared to 13.6 ng/mL in healthy volunteers. Survival was poorer in patients with serum cytochrome levels above 40 ng/mL and the optimal cut-off values for cytochrome *c* in preoperative patients with cancer were 22.7 ng/mL for metastasis, and 22.3 ng/mL for invasion (27). In a cohort of patients with non-small cell lung cancer, more than 13-fold increase in serum cytochrome *c* level was observed after the first cycle of conventional chemotherapy. Assessment of this increase has been proposed as a sensitive apoptotic marker *in vivo*, reflecting chemotherapy-induced cell death burden in patients with non-small cell lung cancer (28).

These aforementioned studies confirmed cytochrome *c* release into the extracellular space and ultimately into the blood in various conditions characterized by cell death. Evidently, the more the cellular or tissue damage, the higher the serum cytochrome *c* level. Thus, cytochrome *c* may be a useful clinical marker for diagnosing and assessing the severity of such pathological entities. Reasonably, detection of high cytochrome *c* level into the circulation concurs with release of various other molecules that serve as DAMPs when found extracellularly, such as mtDNA and FPs. Finally, the release of this universal compound into the extracellular space makes cytochrome *c* an ideal molecule to act as a DAMP.

## Evidence for the Role of Cytochrome *c per se* as a Mitochondrial DAMP

In most of the above studies that confirmed the release of cytochrome *c* into the circulation, the evaluated pathological entities were characterized not only by cell apoptosis and/or necrosis but also by local inflammation. However, there is evidence that serum cytochrome *c* levels increase in pathological conditions characterized by systemic inflammation as well, supporting its possible role as a DAMP.

The prototype of acute systemic inflammation is the systemic inflammatory response syndrome. Serum cytochrome *c* concentrations were markedly increased as measured in intensive care unit patients with systemic inflammatory response syndrome along with or at risk for multiple organ dysfunction syndrome. Mean serum cytochrome *c* concentration was 39.1 ng/mL in patients with acute pancreatitis, 12.09 ng/mL in septic patients, 17.62 ng/mL in patients with septic shock, 2.13 ng/mL in surgical patients, 1.9 ng/mL in patients with severe burns and less than 0.1 ng/mL in control subjects. Cytochrome *c* level correlated well with two representative organ dysfunction scores, APACHE II and multiorgan failure score. Interestingly, cytochrome *c* concentration increased earlier than the multiple organ failure score in the exacerbation phase of the disease and remained high in non-survivors. On the contrary, in the convalescence phase of the disease, cytochrome *c* level decreased sharply in survivors (30). The prognostic value of the cytochrome *c* was also confirmed in acute encephalopathy with multiple organ failure (31).

Chronic hemodialysis could be characterized as a chronic systemic inflammatory condition. Many factors related to uremia *per se*, hemodialysis procedure, complications of chronic renal disease, and therapeutic interventions for their treatment have been associated with the inflammation that characterizes hemodialysis patients. In its turn, chronic inflammation contributes to atherosclerosis, malnutrition, resistance to erythropoietin treatment, and decreased response to vaccination with T-cell dependent antigens that characterize this population (32). Compared to healthy volunteers, in a cohort of free from infections, cancer or autoimmune diseases hemodialysis patients serum cytochrome *c* concentration was found markedly increased. Enzyme-linked immunosorbent assay revealed a serum cytochrome *c* concentration of 1392.88 ± 905.24 pg/mL in hemodialysis patients; whereas in healthy volunteers, it was only 212.95 ± 91.71 pg/mL. As expected, the level of the pro-inflammatory interleukin-6 (IL-6) was also elevated (50.32 ± 35.89 vs. 14.27 ± 6.83 pg/mL). Interestingly, serum concentration of cytochrome *c* was strongly related to serum IL-6 level (*r* = 0.458), suggesting a cause and effect relationship (33). The source of serum cytochrome *c* in hemodialysis patients remains to be elucidated, yet both uremia and hemodialysis procedure may be implicated since they induce white blood cell apoptosis (34, 35).

Apart from clinical evidence for the role of cytochrome *c* as a DAMP, the most valid proof substantiating that role, derived from a controlled experimental study (36). *In vitro* stimulation of mouse spleen cells with exogenous cytochrome *c* resulted in activation of the transcription factor NF-κB along with increased concentrations of pro-inflammatory cytokines and chemokines into the culture medium. *In vivo* hisotopathological signs of arthritis were evident in mice injected intra-articularly with cytochrome *c*, with a synovitis characterized by macrophage antigen 1 positive (Mac-1+) cell infiltration. Mac-1 is present in polymorphonuclear neutrophils, NK cells, and monocytes/macrophages. Depletion of neutrophils and monocytes resulted in abrogation of cytochrome *c*-induced arthritis. This experimental study confirms the role of cytochrome *c* as a DAMP, since its presence in inappropriate compartments elicited an immune response. Interestingly, the authors of this study rigorously tested the used cytochrome *c* for lipopolysaccharide (LPS) contamination and excluded any possible misinterpretation of the results due to an LPS effect. However, since the studies that support the role of cytochrome *c* as a DAMP are not robust, extra studies are required. In addition, further studies are necessary in order to define the PRR(s) that recognizes the cytochrome *c*, the exact type of the responding cells and the exact molecular mechanisms.

## Conclusion

**Figure 1:**
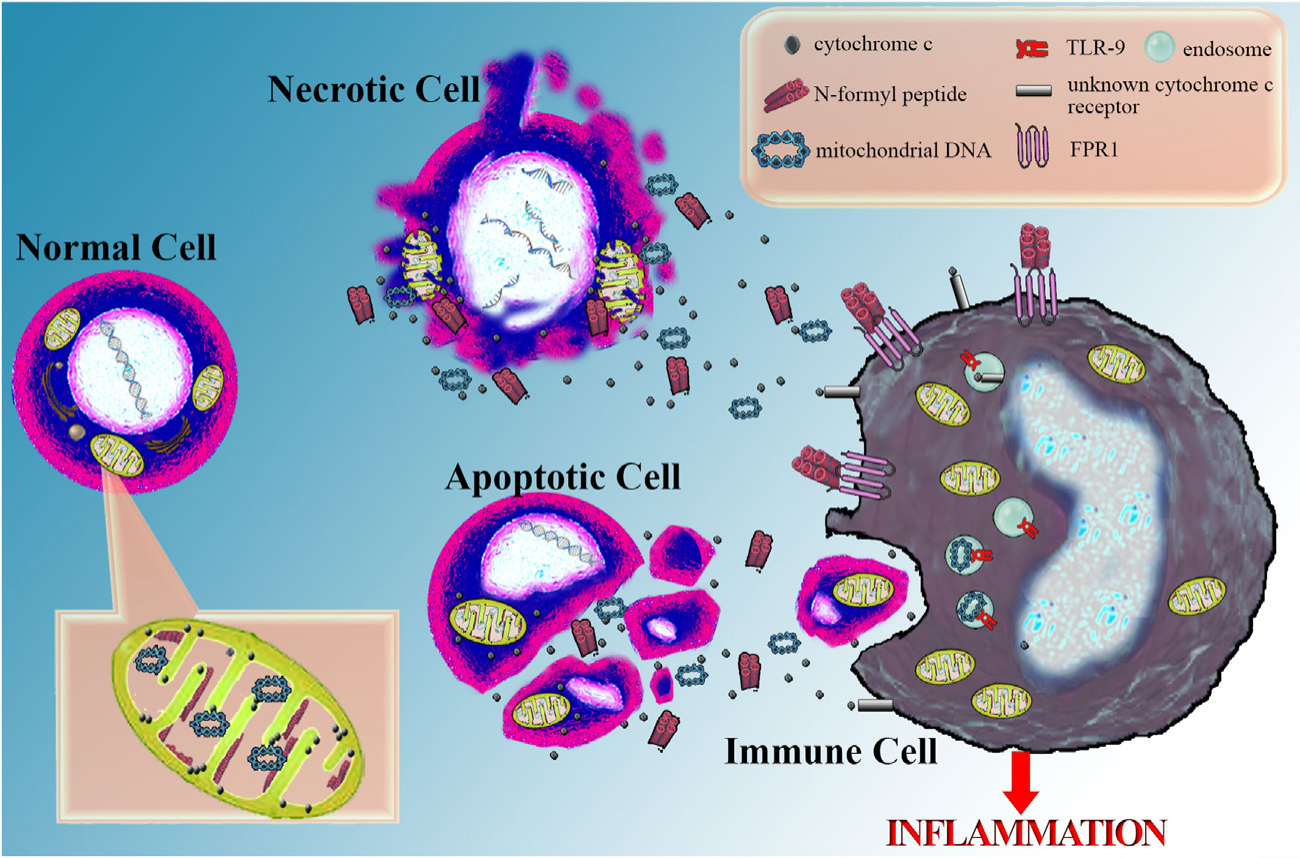
**Cytochrome *c* as a marker of cellular damage and its potential role as a danger-associated molecular pattern**. Serious cellular damage results in cell apoptosis or necrosis. In both cases, cytochrome *c* is released into the extracellular space and can be easily measured in the serum serving as a marker of severe cellular damage and death. Possibly, along with cytochrome *c*, other mitochondrial molecules are also released into the extracellular space. Among them are annotated some well-defined danger-associated molecular patterns (DAMPs), such as mitochondrial DNA, recognized by the toll-like receptor 9 (TLR9) into the endosomes of immune cells and *N*-formyl peptides, sensitized by the formyl peptide receptor 1 (FPR1) on the cell membrane of immune cells. Besides the above DAMPs, which resemble bacterial structures, there is evidence that cytochrome *c*, a universal self-molecule, when is inappropriately located into the extracellular space may behave as a DAMP and elicit an inflammatory response. However, further studies are required for supporting this role for cytochrome *c* and the responsible pattern recognition receptor(s) remain to be discovered.

The conclusions of this mini-review are summarized in Figure 1.

All studies converge to the fact that in case of cell apoptosis or necrosis cytochrome *c* is released from the mitochondria into the extracellular space. Many studies confirmed that serum cytochrome *c* can be easily measured and used for diagnosing and assessing the severity of pathological entities characterized by cell death. However, different studies have shown diverse serum cytochrome *c* levels in various clinical entities and in healthy controls as well. The cut-off that discriminates normal from high-abnormal serum cytochrome *c* values has to be determined before the introduction of such a test into the clinical practice.

The greater the cell death, the higher the concentration of serum cytochrome *c*. Cell death leads to the concurrent release of cytochrome *c* and many other intracellular compounds into the extracellular space with some of them being also DAMPs, such as mtDNA and FPs. Thus, the circulating cytochrome *c* could be considered as an indirect but reliable marker of DAMPs release into the extracellular space and of the consequent systemic inflammation.

Finally, there is evidence for the role of cytochrome *c* as a DAMP. DAMPs are often normal self-molecules found into inappropriate compartments. Consequently, translocation of cytochrome *c* into the extracellular space provides the condition to act as an ideal DAMP. Cytochrome *c* is universally expressed in relatively large amounts in all cells and as such may play the role of a universal DAMP able of alarming the immune system for danger in any type of cell or tissue. In addition, cytochrome *c* is restricted within the mitochondria. Even its exodus into the cytoplasm triggers apoptosis. Thus, there are regulatory mechanisms for keeping cytochrome *c* into the mitochondria, whereas the extracellular compartment is definitely an inappropriate place for it. Nevertheless, the available well-designed experimental studies are sparse and additional studies are required about the role of cytochrome *c* as a DAMP. Certainly, further studies are also required in order to identify the PRR(s) sensitized by cytochrome *c*, the exact type of the responding cells and the exact molecular mechanisms.

## Author Contributions

All authors listed have made substantial, direct, and intellectual contribution to the work and approved it for publication.

## Conflict of Interest Statement

The authors declare that the research was conducted in the absence of any commercial or financial relationships that could be construed as a potential conflict of interest.
